# A Hydration Model to Evaluate the Properties of Cement–Quartz Powder Hybrid Concrete

**DOI:** 10.3390/ma17112769

**Published:** 2024-06-06

**Authors:** Bo Yang, Yao Liu, Xiao-Yong Wang

**Affiliations:** 1 Department of Architectural Engineering, Kangwon National University, Chuncheon-si 24341, Republic of Korea; yangbo@kangwon.ac.kr; 2 Department of Integrated Energy and Infra System, Kangwon National University, Chuncheon-si 24341, Republic of Korea

**Keywords:** cement, quartz powder, fineness, replacement ratio, hydration model, properties prediction

## Abstract

Although quartz powder is a common concrete filling material, the importance and originality of this study lies in the development of a hydration model for quartz powder–cement binary mixtures and the adoption of this model to predict the development of concrete material properties. The purpose of this study is to use this model to promote the material design of environmentally friendly concrete and to elucidate the relationships in the development of the various properties of quartz powder concrete. The method used in this study was as follows: The parameters of the hydration model were obtained through seven days of hydration heat experiments. The hydration heat up to 28 days was also calculated, and the various properties of the concrete were predicted from the heat of hydration. The main findings of this study were as follows: (1) The ultimate hydration heat released per gram of cement for the different quartz powder substitution rates and quartz powder particle fineness was the same, at 390.145 J/g cement, as was the shape index of the hydration model at −1.003. (2) Moreover, through the model calculations, we found that, at the twenty-eighth day of the curing period for the quartz powder specimens with different quartz powder substitution amounts and different fineness, the reaction level of the cement was similar, at 0.963, as were the values of the cumulative heat of hydration, with both at 375.5 J/g cement. (3) The model showed that, in the late stage (28 days) of hydration for quartz powders of different fineness and when the substitution amount was the same, the cumulative heat of hydration over 28 days was similar. (4) The properties of concrete were evaluated using the calculated hydration heat. Overall, the predictive performance of the power and linear functions was similar, with no significant differences being found.

## 1. Introduction

Cement and concrete are among the most commonly used construction materials for infrastructure. In order to achieve the goal of sustainable development and to improve the performance of concrete, quartz powder is increasingly being used in the manufacture of modern concrete [1]. Quartz powder is a by-product of the stone industry. The application of this by-product in the cement industry is part of the circular economy, which improves the resource utilization efficiency of the entire socio-industrial chain, reduces waste emissions, and promotes the overall sustainable development of society [2].

To date, there have been many experimental studies on concrete materials. However, compared with the abundant experimental studies, theoretical studies are limited in the current literature. Hydration models are an aspect of theoretical research. Based on a hydration model, researchers can predict various properties of concrete materials. The hydration of cement is closely related to the composition of the cementitious material. According to the differences in cementitious material components, the research on hydration models so far can be roughly divided into the following categories:

First, there are many hydration models describing Portland cement. Park et al. [3] developed a hydration model for individual cement particles that takes different reaction stages during the cement hydration process into account, such as the initial latent period, the interfacial reaction period, and the diffusion period. The chemically bound water, porosity, and relative humidity were then calculated. In addition, combined with the finite element method, the temperature distribution and temperature development history of concrete during the hardening process were calculated [4]. Maruyama et al. [5] developed a multi-component hydration model that takes the hydration level of the mineral composition of the cement into account and calculates the reactivity of C_3_S, C_2_S, C_3_A, and C_4_AF at different curing times. On the basis of the hydration levels of different mineral components, the average hydration level of the cement was calculated. The model developed by Breugel [6] can simulate the hydration process, the formation of capillary pores, and the connections between cement particles of different sizes [7], thus predicting the development of the cement’s strength [8] and durability [9]. The model developed by Lin and Meyer [10] takes the effects of curing temperature and ambient atmospheric pressure on the hydration rate of cement into account. In oil-well environments, high temperature and high pressure are common environmental conditions. Therefore, Lin and Meyer’s [10] model is well suited to modeling the hydration of oil-well cement. Recently, Zhang et al. [11] developed a model that can predict the evolution of the composition of hydration products with age, which provides good initial conditions for a durability assessment of concrete. This is because durability is closely related to the composition and content of cement hydration products.

Second, hydration models that are suitable for cementitious materials containing admixtures are limited in relation to Portland cement. The model developed by Schutter [12] simulates the reaction of cement and slag. Based on these two reactions, it predicts the amount of hydration heat generated, the average reactivity of the cementitious material, the early creep [13], the heat of hydration, the temperature changes in the concrete [14], and the development of its mechanical properties [15]. The model developed by Bentz et al. predicts the dissolution process, diffusion process, and the reaction process of cement particles during the hydration of cementitious materials [16]. Moreover, it predicts the temperature rise under the adiabatic temperature rise in concrete containing silica fume [17], changes in the chemical composition of the hydration products [18], and the chloride ion diffusion coefficient [19]. The chemical reaction equation developed by Papadakis can predict the influence of silica fume [20] and fly ash [21,22] on the ultimate hydration product, the strength equivalence coefficient, and the durability equivalence coefficient of various mineral admixtures [23]. The carbonation model of concrete materials developed by Papadakis [23,24] has been cited by many researchers [25] and has been verified in the actual use of concrete. The hydration model developed by Maekawa et al. [26,27,28,29,30] takes the hydration exotherm of the mineral composition of cement and the hydration exotherm of mineral admixtures into account and predicts the changes in the mechanical properties and shrinkage of concrete under different mix ratios and different curing conditions [28]. It also predicts the drying shrinkage and various durability properties, such as carbonation [26], chloride ion erosion, and steel corrosion [26]. A coupled analysis of the materials and structures was also performed [26].

However, the aforementioned models do not describe the composition of the hydration products or the composition of the pore solutions. In order to fill this gap, Barbara et al. [31,32,33,34,35,36] developed a series of thermodynamic hydration models. The composition of the hydration products and the pore solutions at different curing ages were calculated [36] with the help of GEMS thermodynamics software (https://cemgems.org/, accessed on 1 May 2024) [31], using the hydration level and the cementitious material composition as the input parameters [35]. In addition, the mechanical and durability properties [34] and the microstructure of the hydration products were also summarized [33]. The inclusion of thermodynamic methods improves the chemical accuracy of hydration models, allowing them to be compared more accurately with experimentally measured results.

On the basis of the above literature survey, we found that the previous models have the following shortcomings: First, in terms of the research object, there has been a lack of hydration models for quartz powder hybrid materials. Quartz powder is widely used in the manufacture of self-compacting concrete and ultra-high-performance concrete [2]. In order to meet the material design needs of the concrete industry, a reliable concrete hydration model that considers quartz powder is necessary. Second, previous models used a large number of complex formulas in their research methods. Objectively speaking, these complex formulas have limited the promotion and use of these models. At present, experimental research is more extensive than theoretical research in the field of cement and concrete materials. What most researchers in experimental science need are simple models with clear concepts [37] and good repeatability rather than complicated models with a large number of parameters. Finally, in terms of prediction objects, previous researchers have mostly focused on the prediction of hydration heat and strength; however, there are other testing methods that can be used in concrete material testing. For example, ultrasonic pulse velocity can be used as a non-destructive testing method for strength, and surface electrical resistivity can be used as a non-destructive testing method for the corrosion resistance of concrete materials [38]. Science researchers and engineering designers have questions about whether hydration models can be used to predict the results of these non-destructive testing methods in a large number of materials.

In order to overcome the shortcomings of previous hydration models, a hydration model for concrete with quartz powder was developed in this study. This model considers the influence of the quartz powder’s substitution rate and particle size. It is also a simple, conceptually clear model with only three parameters. Despite its relative simplicity, the model is comprehensive and was able to systematically predict the heat of hydration, compressive strength, ultrasonic pulse velocity, and surface electrical resistivity of concrete. We believe that the hydration model developed in this study can promote the application of quartz powder in the field of concrete, and the development of concrete material science and new low-carbon concrete materials.

The paper makes several key contributions: Firstly, it proposes a three-parameter hydration model to forecast the long-term hydration heat of a cement–quartz powder binary mixture. This model takes into account the fineness of quartz powder and the amount of quartz powder substitution. Secondly, it predicts the early and long-term mechanical properties of hardening concrete based on the hydration heat. The early mechanical properties can be utilized for construction management, while the long-term properties are valuable for structural design. Lastly, it forecasts the surface electrical resistivity of hardening concrete through the hydration heat, which is an important parameter for measuring the corrosion rate of steel bars. The model introduced in this paper has the potential to be implemented in the field of steel bar corrosion durability.

## 2. Hydration Model and Evaluation of Properties

### 2.1. Experimental Outline

In our previous research [38], experiments were conducted on the effects of the quartz powder substitution amount and of its fineness on the properties of concrete materials. The raw materials that were used in these experiments were as follows: the cement used was Portland cement, and the quartz powder was divided into two particle size levels: small and large. The smaller quartz powder had a particle size range of 1 micron to 3 microns. For these smaller quartz powder specimens, two replacement levels were used: 10% and 20%. The larger quartz powder had a particle size range of 9 microns to 15 microns. For the larger quartz powder specimens, one replacement level was used: 10%. The sand used was standard sand. As shown in Table 1, the mass ratio of water and cementitious materials was 0.5, and, for the mortar specimens, the mass ratio of sand and cementitious materials was 2.5. We measured the isothermal heat release during hydration (from the beginning of stirring to 7 days) using a TAM Air isothermal calorimeter according to ASTM C1702 [39]; measured the compressive strength (at 3, 7, and 28 days) according to ASTM C109 [40]; measured the ultrasonic pulse velocity (at 3, 7, and 28 days) according to ASTM C597 [41]; and measured the surface electrical resistivity (at 3, 7, and 28 days) according to ASTM D3633 [42]. For the hydration heat test, paste specimen was used. And for the compressive strength test, ultrasonic pulse velocity test, and surface electrical resistivity test, mortar specimens were used. In situ photos of the hydration heat test, compressive strength test, ultrasonic pulse velocity, and surface electrical resistivity test are shown in Figure S1a–d, respectively. All the specimens were sealed and cured at a temperature of 20 °C. The general trend of the test results was given a certain replacement ratio; the cumulative hydration heat of 1 g of cement increased as the fineness of the quartz powder was increased. At a certain fineness, as the quartz content was increased, the cumulative hydration heat, compressive strength, ultrasonic pulse velocity, and the surface electrical resistivity decreased.

### 2.2. Hydration Model

Previous experimental studies have shown that quartz powder is a chemically inert filler and has no chemical reaction. It has mainly been found to have dilution and nucleation effects. The dilution effect means that, after quartz powder replaces part of the cement, the mass ratio of the cement and water increases, resulting in an accelerated hydration rate. The nucleation effect means that, as an inert filler, the cement’s hydration products can be formed on the surface of the quartz powder, which accelerates the hydration reaction rate of the cement and increases the heat release corresponding to its unit mass.

**Figure 1:**
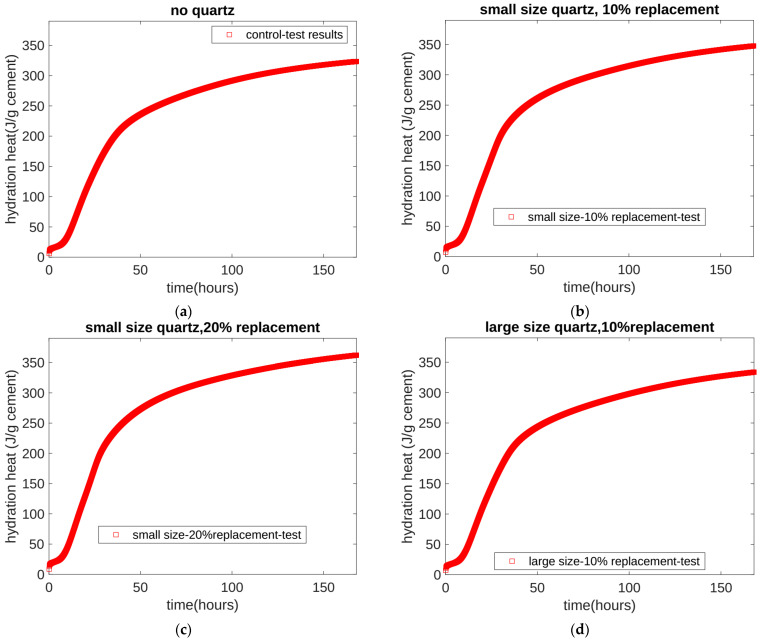
Test results for isothermal hydration heat. (**a**) Test results for the hydration heat of Mix 1. (**b**) Test results for the hydration heat of Mix 2. (**c**) Test results for the hydration heat of Mix 3. (**d**) Test results for the hydration heat of Mix 4.

In the binary mixture of cement–quartz powder, the hydration of the cement played a dominant role, and the physical effect of the quartz powder changed the hydration rate of the cement. In order to better analyze the role of quartz powder, we normalized the measured results of the heat of hydration based on the cement mass. The normalized results are shown as the solid red line in Figure 1. Figure 1 contains four subfigures, and each subfigure contains the experimental results adapted from the study referenced in [38]. Figure 1a does not incorporate quartz powder, and Figure 1b,c includes small particle quartz powder. In Figure 1a–c, the experimental values of the seven-day cumulative heat of hydration can be seen as 323.4, 347.6, and 361.4 J/g cement, respectively. The increments in the hydration heat occurred because the physical effect of quartz powder accelerates cement’s hydration. Figure 1d shows large-particle quartz powder, for which the substitution amount was 10%. In Figure 1b,d, the experimental values of the seven-day cumulative heat of hydration can be seen as 347.6 and 333.5 J/g cement, respectively. In Figure 1b,d, the substitution amount of quartz powder is the same, 10%, but the particle size of the quartz powder is different. We can see that, as the particle size of quartz powder increases, the acceleration effect of the quartz powder on the cement’s hydration became less obvious. In conclusion, based on the experimental results of the seven days of cumulative hydration heat, we found that the physical effect of quartz powder mainly depends on its substitution amount and particle size. As the substitution amount increases and the particle size decreases, the physical effects of the quartz powder become significant.

The three-parameter model is one of the most effective formulas for predicting cement’s hydration level [10]. The three-parameter model can evaluate the properties of hardening cement-based materials [10] such as strength development, heat release, and the formation of hydration products. In order to predict the heat of hydration of cement containing quartz powder, the prediction formula can be expressed as in Equation (1) [10] as follows:

*Q*(*t*) = *Q_CU_* × exp(−*r* × (*t^s^*)) (1)

In this equation, *Q_CU_* represents the heat of hydration released when 1 g of cement is ultimately hydrated; *t* is the hydration time; *Q*(*t*) represents the heat of hydration as a function of time; *r* represents the reaction rate parameter; and *s* represents the shape index of the heat of hydration curve. Equation (1) is not proposed for the first time in this study. In previous studies, Equation (1) has been widely used to predict the development of cement-based material properties, such as the degree of hydration, chemical shrinkage, and chemically combined water [10].

However, it should be noted that previous researchers did not consider the influence of quartz powder when using Equation (1). In this study, we overcame the shortcomings of previous studies by using this equation to simulate the influence of the fineness and substitution amount of quartz powder on cement hydration for the first time.

Based on the experimental results from the seven days of accumulated hydration heat, we performed a regression on the parameters in Equation (1). The parameter regression process can be summarized as follows: First, based on the experimental results of the control specimen, the values of the final heat of hydration *Qcu*, the shape coefficient *s*, and the reaction rate coefficient *r* were obtained. Second, keeping the final heat of hydration *Qcu* and shape coefficient *s* unchanged, the value of the reaction rate coefficient *r* for each mix ratio was obtained.

The regression results are shown in Table 2. They show that, for different quartz powder substitution rates and particle sizes, the parameter value *Q_CU_* corresponded to the complete hydration of 1 g of cement. Moreover, the shape index *s* of the cumulative hydration heat can be seen to be the same: *Q_CU_* = 390.145 J/g cement and *s* = −1.003.

**Figure 2:**
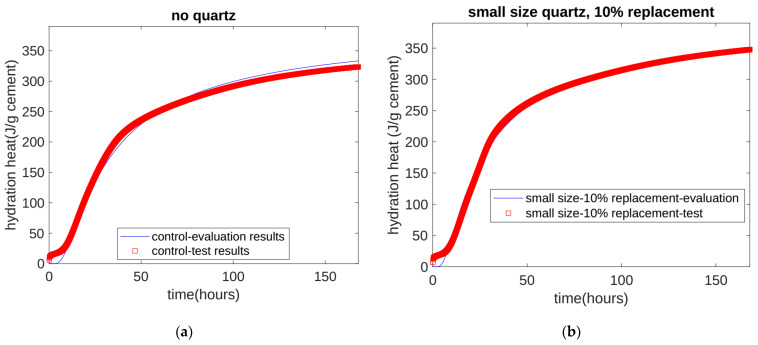

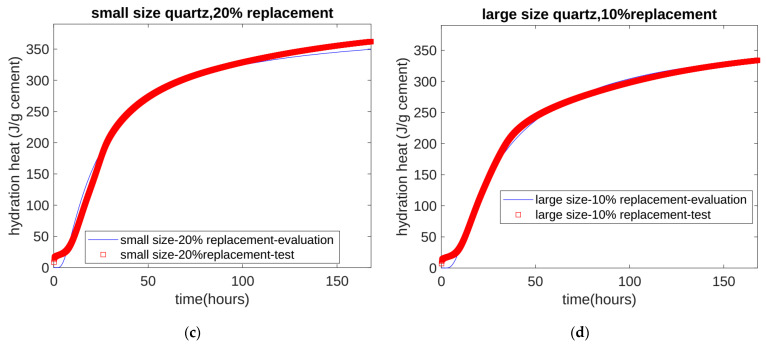
Analysis of isothermal hydration heat. (**a**) Analysis of hydration heat of Mix 1. (**b**) Analysis of hydration heat of Mix 2. (**c**) Analysis of hydration heat of Mix 3. (**d**) Analysis of hydration heat of Mix 4.

In Table 2, *r*1, *r*2, *r*3, and *r*4 are the reaction rate parameters of the mix ratios Mix 1, Mix 2, Mix 3, and Mix 4, respectively. Moreover, *r*1, *r*2, *r*3, and *r*4 were equal to 26.95, 21.39, 18.78, and 25.30, respectively. The decrease in the *r* value means that the rate of the reaction was accelerated. Therefore, we can conclude that, for the mix ratios Mix 1, Mix 2, and Mix 3, as the *r* value decreased (corresponding to the same t value) the cumulative hydration heat increased, which was consistent with the trend of the experimental results shown in Figure 2a–c. For the mix ratios Mix 2 and Mix 4, as the *r* value increased (corresponding to the same *t* value), the accumulated hydration heat decreased, which was consistent with the trend of the experimental results shown in Figure 2b,d. In addition, we noticed that in the initial stage of cement hydration—from the beginning of mixing to approximately 10 h of hydration—the simulated results were slightly smaller than the experimentally measured results. This is because Equation (1) did not consider the initial rapid heat release of the hydration process and the following dormant period.

**Figure 3:**
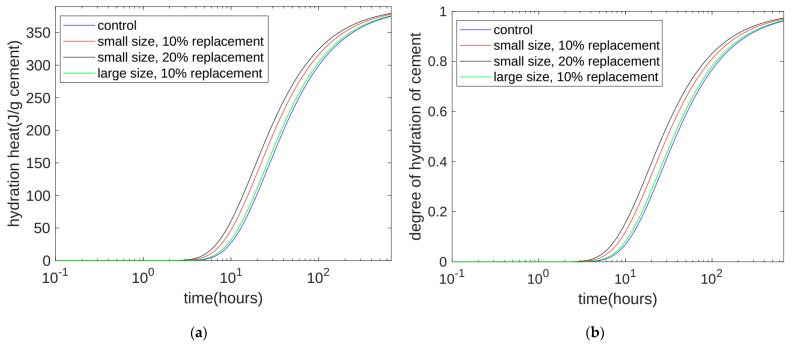
Calculation results of the long-term hydration heat and reaction level of cement. (**a**) Calculation results of the long-term hydration heat. (**b**) Calculation results of the long-term reaction level of cement.

Using Equation (1), we can calculate the heat of hydration for longer periods of time. Because our test period was 28 days, we calculated the heat of hydration from the start of stirring to the 28th day. In order to better observe the difference between each curve, we used logarithmic coordinates to express time. The calculated results, shown in Figure 3a, show that, for a given time point, the order of accumulated hydration heat was Mix 3, Mix 2, Mix 4, and Mix 1. Moreover, in the early stage of hydration, the difference between each heat release curve was large. As time went by and the later stage of hydration was reached, the cumulative heat release of each curve was approximately the same. This result shows that quartz powder mainly plays a physical role, accelerating the rate of the cement hydration reaction, but that it does not change the ultimate heat release per gram of cement.

The hydration level of cement can be defined as the hydration heat release value divided by the maximum value of the hydration heat that can be released: *Q*(*t*)/*Q_CU_*. We can use Equation (1) to calculate the level of hydration. The calculation results for the level of hydration are shown in Figure 3b. The development trend of the hydration level and the trend of hydration heat were very similar. In the early stage, the hydration level increased rapidly. In the later period (28 days), the hydration level of each mix ratio was approximately equal to 0.963. This means that almost all of the cement had been hydrated.

### 2.3. Property Evaluations

#### 2.3.1. Evaluation of Long-Term Hydration Heat

**Figure 4:**
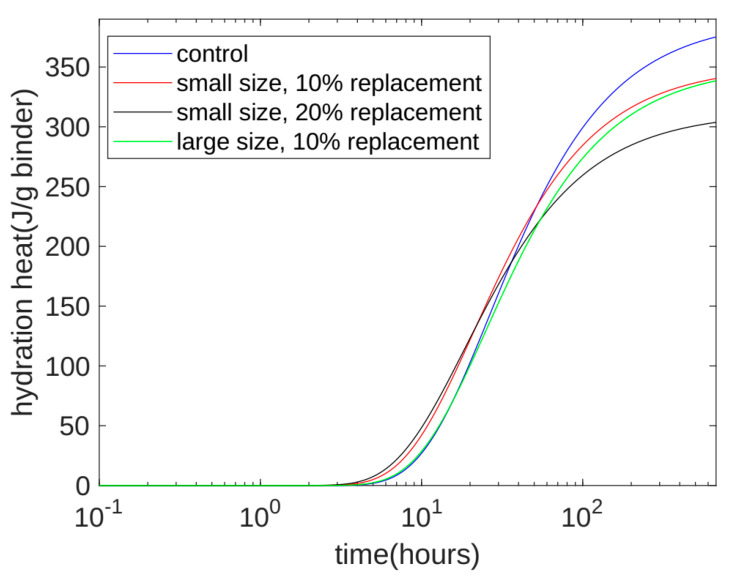
Calculation results of long-term hydration heat of 1 g binder.

Please note that, in Figure 2 and Figure 3, all results for the cumulative heat of hydration are given per gram of cement. In actual concrete property testing—such as compressive strength, ultrasonic pulse velocity, and surface electrical resistivity experiments—most of the experimental results are expressed based on the sum of the masses of cement and quartz powder. In order to analyze the relationship between the heat release and other properties of concrete, we transformed the calculation results in Figure 3a from the cumulative heat of hydration based on each gram of cement to the heat of hydration based on the sum of the mass of the cement and quartz powder. The calculated results for the converted heat of hydration are shown in Figure 4. First, we can see that, at 28 days after hydration, the cumulative heat of hydration values of Mixes 1, 2, and 3 were 374.6, 340.2, and 303.4 J/g binder, respectively. This means that, as the quantity of small-particle quartz powder increased, the cumulative heat of hydration over 28 days decreased. This was mainly due to the diluting effect of the quartz powder on the cement in the later stage, which was similar to the trend of the compressive strength results. Second, we can see that, in the early stage of hydration—from the start of stirring to approximately 30 h—the cumulative heat of hydration of the fine-particle quartz powder was higher than that of the control specimen, which contained no quartz powder. The order of accumulated hydration heat was Mix 3 > Mix 2 > Mix 1. This shows that, in the early stage of hydration, the nucleation of the fine quartz powder played a dominant role. The increasing effect of nucleation on the heat of hydration outweighs the decreasing effect of dilution on the heat of hydration. Third, we can see that, in the late stage (28 days) of hydration, the cumulative heat of hydration for Mix 4 and Mix 2 was approximately the same: both 340 J/g binder. The cement content of Mix 4 and Mix 2 was also the same: both 90%. The difference between the two was that Mix 2 used small-particle quartz powder, while Mix 4 used large-particle quartz powder. The experimental results show that the particle size of the quartz powder mainly affects the rate of the cumulative hydration heat release but has little effect on the total cumulative hydration heat. Fourth, when large-particle quartz powder was used to replace 10% of the cement (Mix 4), in the early stage of hydration, the cumulative hydration heat release was different from that of the mix without quartz powder (Mix 1). The specimens were approximately the same, which shows that, for large-particle quartz powder, when the substitution amount was 10%, the dilution effect and the nucleation effect were present at the same time. The dilution effect caused a decrease in the heat of hydration, and the nucleation effect caused an increase in the heat of hydration. Generally speaking, before approximately 30 h, the reduction in the dilution effect and the increase in the nucleation effect cancel each other out. Compared with Mix 2, in the early stage of hydration, small-particle quartz powder more obviously increased the heat of hydration than large-particle quartz powder.

#### 2.3.2. Evaluation of Compressive Strength Development

**Figure 5:**
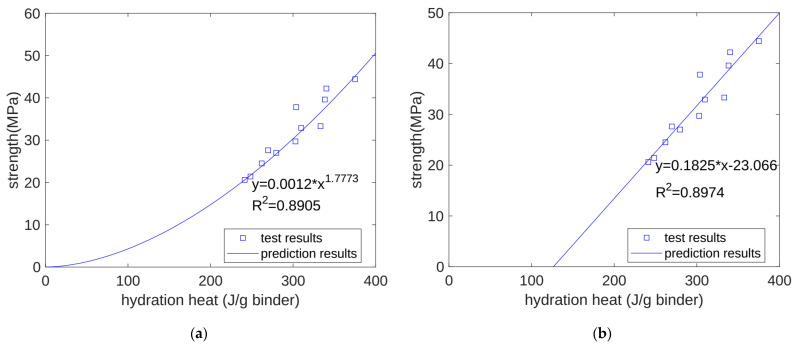
Regression of compressive strength. (**a**) Exponential function regression of strength. (**b**) Linear function regression of strength.

The mechanical properties of concrete materials are closely related to the level of hydration. The heat of hydration is calculated from the level of hydration. Therefore, there may also be a certain correlation between the compressive strength of concrete materials and the heat of hydration. At present, due to the limitations in the accuracy of our hydration heat measuring instrument, it can only measure the heat of hydration for the first 7 days of hydration. However, using our proposed hydration model, we can predict the heat of hydration from the onset of stirring to long-term aging. Based on the calculated results of the heat of hydration at 3 days, 7 days, and 28 days and the measured results of the compressive strength at 3 days, 7 days, and 28 days, we tried to regress the relationship between the compressive strength and the heat of hydration. We used two regression equations: a power exponential function and a linear function. As shown in Figure 5a,b, both the power exponential function and the linear function successfully expressed the relationship between compressive strength and hydration heat, and the regression correlation coefficients were 0.8905 and 0.8974, respectively. In other words, when using both of these functions to regress the relationship between the compressive strength and heat of hydration, there was no obvious difference in the performance of the regression.

#### 2.3.3. Evaluation of Ultrasonic Pulse Velocity Development

**Figure 6:**
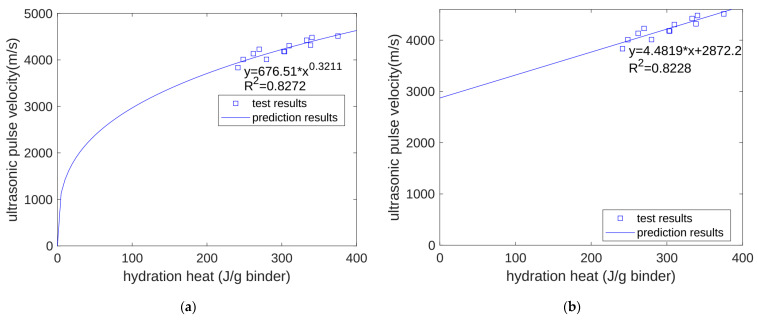
Regression of UPV. (**a**) Power function regression of UPV. (**b**) Linear function regression of UPV.

Ultrasonic pulse velocity is one of the most effective ways to measure solid hydration products in concrete. The content of solid hydration products is also closely related to the hydration level of cement. Therefore, we estimated that there would also be some correlation between the ultrasonic pulse velocity and the accumulated hydration heat. For the ultrasonic pulse velocity, the regression method used was similar to that used for compressive strength. We calculated the cumulative hydration heat at 3 days, 7 days, and 28 days. We experimentally measured the ultrasonic pulse velocity at the same age and performed a regression on the relationship between the two. Like in the regression results for compressive strength, the regression effect of the power function (shown in Figure 6a) was similar to the regression effect of the linear function (shown in Figure 6b). The regression correlation coefficients of the two were 0.8272 and 0.8228, respectively.

#### 2.3.4. Evaluation of Surface Electrical Resistivity Development

**Figure 7:**
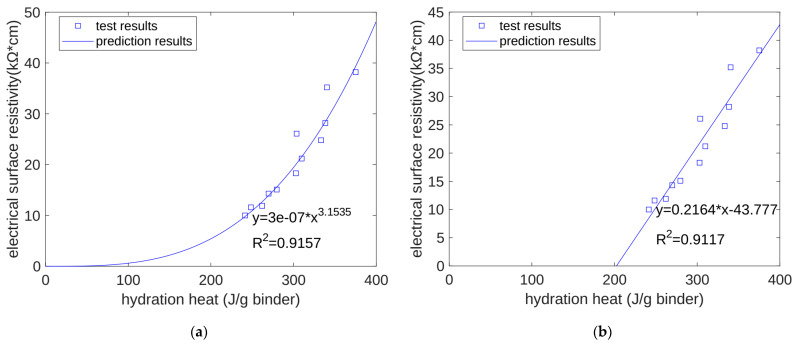
Regression of electrical resistivity. (**a**) Power function regression of electrical resistivity. (**b**) Linear function regression of electrical resistivity.

As cement hydration proceeds, the capillary water of concrete is continuously consumed, the porosity continues to decrease, and its surface electrical resistivity increases. The surface electrical resistivity of concrete is one of the most important indicators in measuring the corrosion resistance of concrete materials. The basic cause of changes in the concrete surface electrical resistivity is the hydration of the cement. Therefore, we speculated that there would be a correlation between the surface electrical resistivity of the concrete and its accumulated hydration heat. For the surface electrical resistivity, the regression method used was also similar to that used for compressive strength. We used a power function and a linear function to regress the relationship between the surface electrical resistivity of the concrete and its accumulated hydration heat. We found that the regression correlation coefficients of the power function (shown in Figure 7a) and the linear function (shown in Figure 7b) were 0.9157 and 0.9117, respectively. In other words, for the resistivity of concrete, the regression functions of the power and linear functions were similar, with no obvious difference being found.

## 3. Summary of Hydration Model

First, for the different mix ratios, the coefficients Q_CU_ and s in the hydration model were completely consistent, but the reaction rate coefficient r was different. This was not an accidental mathematical coincidence but the real mechanism of the cement hydration process in the binary system of cement and quartz powder. Quartz powder mainly plays a physical role in cement hydration: it has a dilution effect and nucleation effect. In this study, we took the dilution effect into account by normalizing the measured heat of hydration to the cement mass. We took the nucleation effect into account through the different reaction rate parameters. As the size of the quartz powder particles decreased and the substitution amount of quartz powder increased, the nucleation effect became more obvious.

Second, due to limitations in instrument accuracy, the cumulative heat of hydration of cement can only be measured within approximately seven days after the start of mixing. After more than seven days, the machine cannot accurately measure the heat of hydration because the heat release of hydration is too low. However, the model developed in this study can predict the heat of hydration from the start of stirring to seven days and can also calculate the heat of hydration beyond seven days. The calculated age can be determined according to the user’s requirements. In other words, the present model can help to overcome the accuracy limitations of traditional heat-of-hydration instrumentation. If the measurement hardware device of the traditional hydration heat machine is combined with the software device of the hydration model that was developed in this study, a wider range of functions could be obtained.

Third, the hydration process of cement is a complex process. The thermal aspect of this process is the release of hydration heat, the mechanical aspect is the increase in compressive strength and ultrasonic pulse velocity, and the pore structure aspect is the increase in surface electrical resistivity. In other words, the heat of hydration, compressive strength, ultrasonic pulse velocity, and resistivity are all manifestations of the same process, but from different angles. Using the same metric, such as the heat of hydration based on the sum of the cement and quartz powder masses, it is possible to evaluate the development of other properties, thus predicting the compressive strength, ultrasonic pulse velocity, and resistivity at different ages.

## 4. Comparison with Previous Models and Limitations of Proposed Model

### 4.1. Comparison with Previous Models

First, Bentz developed a model that could be used to simulate the hydration of concrete containing limestone powder fillers [18]. This model is based on the CEMHYD3D software package (https://www.nist.gov/services-resources/software/cemhyd3d, accessed on 1 May 2024). The program functions of this software package are relatively comprehensive and can simulate the microstructure and the generation of hydration products during the hydration reaction. Second, Maekawa and Ishida’s model also considered the effect of limestone powder on cement hydration [26]. In addition, their model also took the effects of other mineral admixtures into account, such as the reaction of fly ash and slag. Based on the hydration model, they developed Ducom software (https://www.comse.co.jp/english/analysis/technology4.html, accessed on 1 May 2024) to predict the early performance and durability properties of concrete. Finally, the model proposed by Breugel considered the impact of cement’s particle size distribution on its hydration rate and pore structure [6,8], and the interaction among the cement particles during the hydration process. Based on this hydration model, the Breugel research team developed the HYMOSTRUC software tool (https://dianafea.com/numerically-simulated-pore-structure-and-its-potential-application-in-high-performance-concrete/, accessed on 1 May 2024). Compared with these software packages, which have now existed for many years [6,8,18,26], the main advantages of the model proposed in this article are that the calculation formula of the model is simple, the concept is clear, and it is convenient for the majority of experimental researchers to use. However, its main limitation is that the function of the hydration model is relatively limited.

### 4.2. Limitations of Proposed Model

The limitations of the model are mainly related to its material and curing environment aspects. At the material level, this model does not consider other reactive mineral admixtures, such as silica fume, slag, and fly ash [26]. In terms of the curing environment, this model does not consider the effect of temperature; temperature can accelerate the hydration of cement, improve its early-age strength, and impair its late-age strength [26].

## 5. Conclusions

This study developed a hydration model for cement and quartz powder binary concrete materials, considering the influence of the substitution amount of quartz powder but also the influence of the quartz powder’s fineness. The prediction results of this model are summarized as follows:

1. Based on the seven-day accumulated hydration heat, the parameters of the three-parameter hydration model were obtained through regression. We found that for different quartz powder substitution rates and quartz powder particle fineness, the ultimate hydration heat release value per gram of cement was the same at 390.145 J/g cement, and the shape index of the hydration model was also the same at −1.003. For Mix 1, Mix 2, Mix 3, and Mix 4, the hydration rate parameters were 26.95, 21.39, 18.78, and 25.30, respectively. As the substitution amount increased or as the particle size of quartz powder decreased, the hydration rate parameter decreased, and the hydration rate of the cement accelerated.
2. Currently, using isothermal hydration heat can only measure the hydration heat from the beginning of stirring to seven days of age. However, our hydration model can calculate the heat and level of hydration over longer periods of time. After the model’s calculation, we found that at 28 days of the curing period, for all the specimens, the reactivity of the cement was similar, with both at 0.963. The values of the cumulative heat of hydration were also similar, with both at 375.5 J/g cement. This shows that the quartz powder mainly plays a physical role by changing the rate of the hydration reaction, but it does not change the ultimate extent of the hydration reaction.
3. The calculation results show that, at approximately 30 h (in the early stage of hydration), the cumulative heat of hydration value for the specimens with fine quartz powder was higher than that for the control specimens. This could be because the nucleation effect of quartz powder played a dominant role. For the coarse quartz, the value of the accumulated hydration heat of the powder specimen was similar to that of the control specimen, which may be due to the acceleration of the nucleation effect and the deceleration of the dilution effect canceling each other out.
4. The calculation results show that, in the late stage of hydration (28 days), as the substitution amount of quartz powder increased, the value of the cumulative heat of hydration decreased. This is because the dilution effect of the quartz powder played a dominant role. For quartz powders of different fineness, when the substitution amount was the same, the cumulative heat of hydration over 28 days was similar. This shows that the fineness of the quartz powder mainly affects early hydration and has a limited influence on later hydration.
5. We predicted the properties of concrete at different ages based on the heat of hydration per gram of cementitious material. For compressive strength, when using a power function and a linear function for regression, the correlation coefficients were 0.8905 and 0.8974, respectively. For ultrasonic pulse velocity, when using a power function and a linear function for regression, the correlation coefficients were 0.8272 and 0.8228, respectively. For surface electrical resistivity, when using a power function and a linear function for regression, the correlation coefficients were 0.9157 and 0.9117, respectively. Overall, the predictive performance of the power and linear functions was similar, with no significant difference being found.

## 6. Recommendations for Future Work

In future research, the following work should be carried out:

1. First, the effects of other reactive admixtures, such as silica fume, slag, and fly ash, should be considered in the hydration model. These reactive admixtures are more widely used in the concrete industry than quartz powder.
2. Second, the effect of temperature in the hydration model and the strength prediction model should be considered, such as the effect of temperature on the hydration reaction rate and compressive strength. This is important, as the development of cement-based material properties is different in hot weather and cold weather.
3. Third, in terms of durability prediction, methods to predict various durability properties based on the hydration degree, such as carbonation, chloride ion penetration, and steel corrosion, should be considered. The proposed model only evaluates properties before 28 days; durability beyond 28 days should be studied in the future.

## Figures and Tables

**Table 1:** Relative mass ratios of specimens.

	Cement (%)	Quartz (%)	Water (%)	Sand (%)
Mix 1	100	0	50	250
Mix 2 (small size)	90	10 (small size)	50	250
Mix 3 (small size)	80	20 (small size)	50	250
Mix 4 (large size)	90	10 (large size)	50	250

**Table 2:** Parameter values of the hydration model.

*Q_CU_*	*r*1 (Mix 1)	*r*2 (Mix 2)	*r*3 (Mix 3)	*r*4 (Mix 4)	*s*
390.145	26.95	21.39	18.78	25.30	−1.003

## Data Availability

The original contributions presented in the study are included in the article/Supplementary Materials, further inquiries can be directed to the corresponding authors.

## Supplementary Materials

The following supporting information can be downloaded at: https://www.mdpi.com/article/10.3390/ma17112769/s1, Figure S1: In situ photos of tests.
