# Supplementary material for: A Hydration Model to Evaluate the Properties of Cement–Quartz Powder Hybrid Concrete
Source: Materials (Basel). 2024 Jun 6;17(11):2769. doi: 10.3390/ma17112769 (PMC11173435; doi:10.3390/ma17112769)
Supplement: Supplementary file 1 [file materials-17-02769-s001.zip › materials-3040356-supplementary.pdf]

Supplementary Materials

In situ photos of the hydration heat test, compressive strength test, ultrasonic pulse velocity, and surface electrical resistivity test are shown in Figure S1a, S1b, S1c, and S1d, respectively.

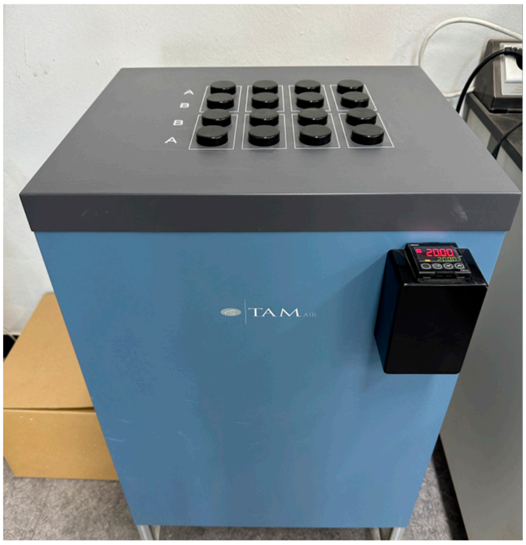

(a) Hydration heat test photos

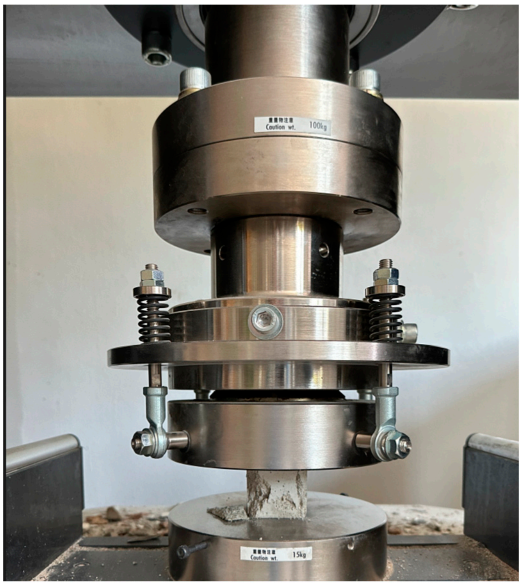

(b) Compressive strength test photos

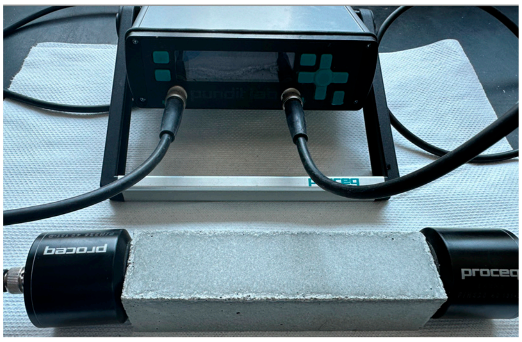

(c) Ultrasonic pulse velocity test photos

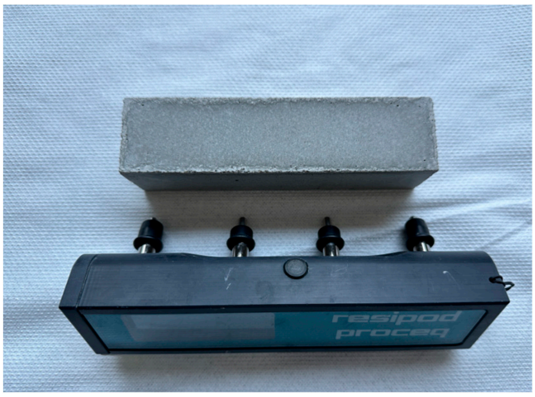

(d) Surface electrical resistivity test photos

Figure S1. In situ photos of tests.
